# The Lessons of Beri-Beri

**Published:** 1901-11-30

**Authors:** 


### THE LESSONS OF BERI-BERI.

At the last meeting of the Epidemiological Society, Dr. Patrick Manson entered very fully into the question of the etiology of beri-beri, and although it is unfortunately true that this question still remains unsolved, yet there was much in what he said which was very suggestive, not only in relation to beri-beri itself, but also in regard to the causation of various other diseases. To those of us who practise in this country beri-beri may perhaps appear a matter of small interest, but the extraordinary gravity of the disease in regions where it prevails may be judged of from the statement made by Dr. Manson that in some plantations over 75 per cent. of the coolies have been killed off by beri-beri in a single year. Two theories are at present held as to the etiology of the disease, viz., the dietetic and the microbic. Among those who have urged the dietetic theory, those have seemed to have most reason on their side who have attributed the disease to prolonged nitrogen starvation, owing to the long-continued use of a uniform rice diet. In proof of this theory some very striking cases have been collected in which after an improvement has been made in the diet the disease, previously very prevalent, has rapidly disappeared. On the other hand, this theory fails to explain all the facts, for numerous examples can be brought forward which show that the disease may prevail among populations whose diet has been specially devised so as to avoid the defects to which the origin of beri-beri has been attributed, and there are also many instances on record of Europeans enjoying a liberal diet falling victims to the disease. As to the germ theory, Dr. Manson, although saying that it is the more plausible of the two, adds that whether the germ produces its morbid effects while proliferating in the human body, or whether it acts indirectly by producing outside the body a toxin which, on being ingested or otherwise absorbed, acts on the nerves, it is impossible to say. On the whole, Dr. Manson inclines to the latter hypothesis, namely, that beri-beri is purely an intoxication, produced by a toxin elaborated by a germ whose nidus is located outside the human body, and "that in this respect beri-beri is on all fours with alcoholism, the germ of which is the yeast plant, the nidus solutions of sugar, the toxin alcohol, and, to complete the parallel, the pathological effect a peripheral neuritis." That the disease is due to a living germ is proved by the facts that (*a*) the cause can be transported from place to place, and therefore cannot be of a climatic or meteorological nature; and (*b*) that when so transported it can multiply and spread, and therefore cannot be of an inorganic nature. The great question is where does the germ live and grow and produce the toxin by which the disease is caused? In answer to this it can at least be shown that certain ships become infected with beri-beri, and if ships why not houses and localities? There is a marked tendency among those who have studied this subject to regard rice as at least the favourite nidus of the disease, but, at any rate, whatever the exact medium in which the poison is distilled, the malady may be regarded as a "place disease," and it is much to be hoped that the expedition which has gone out to study its etiology will be able to isolate the organism by which it is caused and to discover the favourite media in which it grows, and by aid of which it becomes endemic in certain localities. We need hardly point out what an important subject is opened up for inquiry by the suggestive remarks of Dr. Manson. Who is to say what maladies and what conditions of deteriorated health may not arise from the absorption by the human body of the toxins produced by micro-organisms which have their locale in the food, the drink, the raiment, and the houses of mankind? We already know something of ptomaine poisoning, of alcoholism, of pellagra, of ergotism. Now we are told about beri-beri, but who shall say where is the end of the list of diseases which may be called zymotic in that they are due to "germs," but the germs of which are extra corporeal micro-organisms?

